# The Effect of Leptin Receptor Gene Polymorphisms (R223Q and P1019P) in Susceptibility to Polycystic Ovarian Syndrome in Kurdish Women 

**DOI:** 10.22074/IJFS.2021.6197

**Published:** 2021-03-11

**Authors:** Rozita Naseri, Elahe Barzingarosi, Maryam Sohrabi, Yosra Alimoradi, Mostafa Cheraghian Fard, Cyrus Jalili

**Affiliations:** 1 Internal Medicine Department, Imam Reza Hospital, Kermanshah University of Medical Sciences, Kermanshah, Iran; 2 Fertility and Infertility Research Center, Health Technology Institute, Kermanshah University of Medical Sciences, Kermanshah, Iran; 3 Department of Anatomical Sciences, Medical School, Kermanshah University of Medical Sciences, Kermanshah, Iran; 4 Medical Biology Research Center, Health Technology Institute, Kermanshah University of Medical Sciences, Kermanshah, Iran

**Keywords:** Kurdish Population, *LEPR*, Leptin Receptor, Obesity, Polycystic Ovary Syndrome

## Abstract

**Background:**

Polycystic ovary syndrome (PCOS) is the known endocrinopathy disorder in the reproductive phase of women’s life. More than half of the women with PCOS suffer from obesity which impacts the ovarian functions by leptin levels. Here the R223Q and P1019P polymorphisms of leptin receptor (*LEPR*) gene were examined in PCOS patients of Kurdish women from west of Iran.

**Materials and Methods:**

In this case-control study, one hundred women with PCOS and 100 healthy women bearing similar age range were selected based on Rotterdam diagnostic criteria. Polymerase chain reaction-restriction frag- ment length polymorphism (PCR-RFLP) method was used to genotype polymorphisms *LEPR* (R223Q and P1019P), by respectively the BsaWI and NcoI restriction enzymes. Pearson’s chi-square (χ2) test was used to analyze the vari- ation in genetic distributions and unconditional logistic regression model was used to calculate the odds ratio (OR; 95% CI).

**Results:**

Genotype frequencies of the R223Q and P1019P polymorphisms showed significant difference between the patients with PCOS compared to the controls. G allele (R223Q) reduced the risk of PCOS about 0.49-fold (P<0.001). While, T allele (P1019P) increased the risk of PCOS 2.69-fold (P<0.001).

**Conclusion:**

It can be concluded that the R223Q and P1019P polymorphisms showed a significant association with PCOS susceptibility risk. It seems that G allele (R223Q) with reducing OR had a protective effect on this syndrome, while T allele (P1019P) with increasing OR was a risk factor for PCOS.

## Introduction

Polycystic ovary syndrome (PCOS), as a heterogeneous disorder, is yet unexplained and its etiology has uncovered. It is the most common endocrinopathy disorder in the reproductive phase of women life (1) that affects nearly 6-8% of women in this phase (2). The phenotype of PCOS is described by chronic oligoanovulation, hyperandrogenism and polycystic ovarian morphology (3). This complex multi-system syndrome is also associated with hirsutism, insulin resistance and obesity (4).

Besides, as a major cause of infertility, PCOS is often associated with metabolic syndrome characteristics, such as insulin resistance, chronic proinflammatory state, dyslipidemia, increased central or visceral adiposity, etc. The large overlap between features of PCOS and metabolic disorder suggests that dysregulation in the function of adipose tissue contributes to some metabolic dysregulation of PCOS patients, similar to what happens in metabolic disorders (5). Obesity, leading to fat content accumulation in the body especially in adipose tissue, can be considered as a metabolic illness (6, 7).

Adipose tissue by secreting the numerous hormones plays an important role in the metabolism of lipid and carbohydrate, regulation of energy homeostasis, as well as sensitivity to insulin. Cytokines or cytokine-like molecules, including leptin, are the major proportion of these secreted factors which play critical role in immunomodulatory effects and inflammatory responses (5, 8). 

Leptin, which is dysregulated in obesity, is a polypeptide hormone with 16 kDa weight, produced mainly by white adipose tissue and secreted by lipocytes: leptin can affect the immune processes, hematopoiesis, angiogenesis and reproduction (9). In addition, leptin is involved in the metabolism and function of certain tissues (10).

A significant percentage of patients with PCOS have both reproductive dysfunction and obesity (11). Obesity which modify insulin sensitivity and dynamics of gonadotropin is also associated with ovulation disorders (12). It has been suggested that obesity impacts ovarian functions in patient with PCOS, in part, due to the enhanced intra-follicular levels of leptin and it may cause comparative resistance to gonadotropins (13). There are several polymorphic genes in leptin regulation. Single nucleotide polymorphism (SNP) in the leptin and leptin receptor (LEPR) have been studied as factors that may be associated with PCOS and obesity, which is reported in more than half of the women with PCOS (14-16).

So, the aim of present study was to investigate association of PCOS with two important SNPs in the *LEPR* gene (R223Q and P1019P) within the Kurdish women population from west of Iran.

## Materials and Methods

### Subjects

In this case-control study, overall 200 Kurdish women (Kermanshah province, west region of Iran) including 100 women with PCOS and 100 healthy women were examined. Selection of the patients and controls were verified by a gynecologist in accordance to the revision of Rotterdam diagnostic criteria (15). Accordingly, to consider as PCOS patient, each case had to have, at least, two of the following three criteria. Participants were informed about the purpose of project and signed the informed consent (according to the Helsinki II declaration).

This project was also approved by Ethical Committee of Kermanshah University of Medical Sciences (IR.KUMS. REC.1397.249).

### DNA extraction

DNA extraction from samples (1 ml whole blood) was carried out according to the Moradi et al. (17) method. Integrity of the extracted DNA were distinguished by agarose gel electrophoresis and NanoDrop spectrophotometer (Thermo Fisher, USA) was used to determine concentration of DNA.

### Genotyping

**Fig.1:**
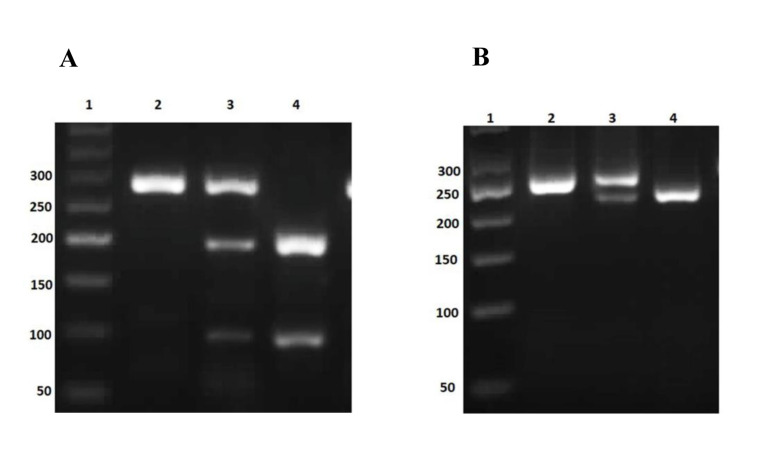
Agarose gel electrophoresis (2%) for PCR product of gene polymorphisms.** A. **PCR product of *LEPR* gene polymorphism (R223Q) on 2% agarose gel. Lines (1) 50 bp DNA ladder, (2) a homozygous individual (AA) with 279 bp band, (3) a heterozygous individual (AG) with 279 bp, 193 bp and 86 bp bands and (4) homozygous individuals (GG) with 193 bp and 86 bp bands. **B.** PCR product of *LEPR* gene polymorphism (P1019P) on 2% agarose gel. Lines (1) 50 bp DNA ladder, (2) a homozygous individual (CC) with 253 bp band, (3) a heterozygous individual (CT) with 253 bp and 223 bp bands and (4) homozygous individuals (TT) with 223 bp band. PCR; Polymerase chain reaction.

Analysis of *LEPR* gene polymorphisms (R223Q and P1019P) were done using polymerase chain reaction (PCR) amplification, followed by restriction fragment length polymorphism (RFLP) techniques. PCR assay was performed in a total volume of 25 µl. It was including 2.5 μl of 10X PCR buffer, 20 pmol of each primer (Table 1), 1.5 mM MgCl_2_ , 0.2 mM dNTPs, 1 Unit Taq DNA polymerase (SinaClonBioScience Co., Iran) and 100 ng isolated DNA as PCR template. The defined condition for thermal cycler included an initial denaturation at 95°C for 2 minutes, 30 cycles of denaturation at 95°C for 30 seconds, annealing at 60°C (*LEPR* R223Q)/53°C (*LEPR*P1019P) for 30 seconds, extension at 72°C for 40 seconds, and then a final extension at 72°C for 5 minutes. After amplification, about 10 µl PCR products were subjected to overnight digestion with 1-2 units of restriction enzymes. Products of the digestion were visualized by 2% agarose gel stained with GelRED under ultraviolet light. In the case of R223Q genotyping, BsaWI (Thermo Fisher, USA) at 60°C produced a 279 bp band (AA genotype), 279 bp, 193 bp, and 86 bp bands (AG genotype), or193 bp and 86 bp bands (GG genotype, Fig.1A). For P1019P genotyping, NcoI (Thermo Fisher, USA) at 37°C produced a 253 bp band (CC genotype), 253 bp and 223 bp bands (CT genotype) or 223 bp band (TT genotype, Fig.1B). 

**Table 1:** The primers used for detection of R223Q and P1019P polymorphisms


NCBI rs#	SNP	Primer sequences (5ˊ-3ˊ)	Method of detection

rs1137101	R223Q	F: ttgtgaatgtcttgtgcct	RFLP-PCR
		R: agaagccactcttaataccc	
rs1805096	P1019P	F: cagatcttgaaaagggttct	RFLP-PCR
		R: tcccatgagctattagagaaagaatccttcca	


SNP; Single nucleotide polymorphism, RFLP; Restriction fragment length polymorphism, and PCR; Polymerase chain reaction.

### Statistical analysis

Statistical analyses were done by SPSS software (V.16). Pearson’s chi-square (χ2) test was utilized to analyze the variation in genetic distributions and unconditional logistic regression model for calculating the odds ratio (OR; 95% CI) and interaction between R223Q and P1029P polymorphisms were used. P<0.05 was considered statistically significant.

## Results

In this study, we examined 100 patients with PCOS and 100 normal controls. Frequency of the patients with PCOS were in Hardy Weinberg equilibrium (P=0.15 for R223Q, P=0.49 for P1019P). There was no significant difference in terms of ages between PCOS patients and controls (the mean age was 28.34 ± 4.58 years for patients and 27.09 ± 5.19 years for controls, P=0.07). Although in terms of body mass index (BMI), it was significantly different (29.14 ± 2.68 for patients and 23.05 ± 2.48 for controls, P<0.001).

The results of this study indicated that genotypic frequency of the R223Q polymorphism was significantly different in the patients with PCOS compared to the controls (Table 2). For R223Q polymorphism, in comparison with controls, AG genotype might associate with 0.41-fold decreased risk of PCOS (P=0.01). G allele also reduced risk of PCOS approximately 0.49-fold (P<0.001). Genotype frequency of P1019P polymorphism was significantly different between the patients with PCOS and the controls. For P1019P polymorphism, in comparison with the controls, TC genotype might associate with 2.43-fold increased risk of PCOS (P=0.007) T allele also increased the risk of PCOS approximately 2.69-fold (P<0.001, Table 3). Study the interaction of D151A and R453Q genotypes showed 0.51-fold decrease in the risk of PCOS (P=0.02) for the carriers ofD151A (AA genotype) and R453Q (AG or AA genotypes), but there was no significant correlation between other combinations of the D151A and R453Q genotypes and risk of PCOS (Table 4).

In order to investigate the association of the gene polymorphisms with BMI, and age, the total participants (patients and healthy controls) were divided into two groups: in terms of BMI (≤25 and >25) and age (≤30 and >30 years old). Results showed that the frequency of genotypes (R223Q/P1019P) had no significant difference in the subgroups divided by age and BMI (Table 5).

**Table 2:** Frequency of R223Q genotypes and alleles


Frequency	Controlsn (%)	Patientsn (%)	Odds ratio (95% CI)	P value

Genotypes				
AA	17 (17)	37 (37)	1	
AG	59 (59)	53 (53)	0.41 (0.21-0.81)	0.01
GG	24 (24)	10 (10)	0.19 (0.075-0.48)	<0.001
AG+GG	83 (83)	63 (63)	0.35 (0.18-0.67)	0.001
Alleles				
A	93	127	1	
G	107	73	0.49 (0.33-0.74)	<0.001


Overall χ2 = 13.494. CI; Confidence interval.

**Table 3:** Frequency of P1019P genotypes and alleles


Genotypes	Controlsn (%)	Patientsn (%)	Odds ratio (95% CI)	P value

P1019P				
CC	80 (80)	59 (59)	1	
TC	19 (19)	34 (34)	2.43 (1.29-4.66)	0.007
TT	1 (1)	7 (7)	9.49 (1.14-79.24)	0.03
TC+TT	20 (20)	41 (41)	2.78 (1.48-5.23)	0.001
Alleles				
C	179	152	1	
T	21	48	2.69 (1.54-4.7)	<0.001


Overall χ2 = 13.494. CI; Confidence interval.

**Table 4:** Interaction between genotypes of R223Q and P1019P onfidence interva


R223Q	P1019P	Odds ratio (95% CI)	P value

AA	CC	1.63 (0.92-2.85)	0.089
AG+GG	CC	2.14 (0.77-5.94)	0.14
AA	TC+TT	0.51 (0.28-0.9)	0.02
AG+GG	TC+TT	0.73 (0.24-2.19)	0.58


CI; Confidence interval.

**Table 5:** Association of age and BMI with the genotypes of R223Q and P1019P polymorphisms


SNP	Genotype	BMI		Odds ratio (95% CI)	P value	Age (Y)		Odds ratio (95% CI)	P value
		≤25	>25			≤30	>30		

R223Q	AA	50	62	1		41	13	1	
	AG	30	24	0.64 (0.33-1.24)	0.18	83	29	1.1 (0.52-2.34)	0.8
	GG	21	13	0.49 (0.28-1.09)	0.08	22	12	1.72 (0.67-4.4)	0.25
P1019P	CC	54	75	1		92	49	1	
	CT	25	38	1.09 (0.59-2.02)	0.77	31	20	1.2 (0.62-2.34)	0.56
	TT	2	6	2.16 (0.42-11.11)	0.36	5	3	1.12 (0.25-4.9)	0.87


SNP; Single-nucleotide polymorphism, BMI; Body mass index, and CI; Confidence interval.

## Discussion

According to our best knowledge, as a novel study in an Iranian population, we evaluated the association between PCOS and two *LEPR* gene polymorphisms (R223Q and P1019P). Here we showed that both variants of *LEPR* (R223Q and P1019P) are related to PCOS susceptibility. Additionally, it seems that G allele (R223Q) has a protective effect on this disease and reduces the corresponding risk of PCOS, while T allele (P1019P) increases the corresponding risk. In addition, based on our results, it seems there is no correlation between these polymorphisms with BMI and age.

Increasing evidences indicated that there was an overlap among the obesity and other metabolic disorders, such as metabolic syndrome, diabetes mellitus and PCOS (18). Villa and Pratley (5) by comparing gene expression profiles in adipose tissue of patients with PCOS and normal control group, showed different dysregulated genes in several ontological classes. They found dysregulated genes were involved in immune function, cell growth, insulin signaling, lipid metabolism and metabolic syndromes. On the other hand, researchers believe that the strong association between obesity and PCOS can refer to the relation between PCOS and obesity-susceptibility variants (19).

Bioactive cytokines and adipokines, including the resistin, adiponectin and leptin, are released from fat tissue (20). It was suggested that there is an association between leptin dysregulation and the onset of obesity as well as the obesity-related pathologies such as PCOS (21, 22). Leptin has important role in the physiology of reproductive system. It has intricate interactions at the hypothalamic-pituitary-gonadal axis such as inhibitory actions at the gonads and stimulatory effects at the pituitary and hypothalamus (23). In total, it is suggested that leptin and its receptor (LEPR) may contribute to the insulin metabolism, energy homeostasis and ovarian androgen synthesis related to PCOS (24).

SNPs of the LEPR gene have been investigated in various local populations and different diseases like breast cancer, non-small-cell lung cancer, oral squamous cell carcinoma, diabetic macroangiopathy and essential hypertension (25- 28). However, few studies have addressed the relationship of *LEPR* variants and PCOS. Two known polymorphisms of *LEPR* are R223Q and P1019P. Although the biological functions of these two polymorphisms are not fully understood, it has been reported that R223Q and P1019P are respectively found in the extracellular and intracellular regions of the receptor and separately involved in cytokine motifs and ligand binding. Similar to our study, a casecontrol study in a Korean population reported significant association between the variants of *LEPR* (R223Q and P1019P) and risk of PCOS (29). While a case-control study in Finland reported no association between the *LEPR* variants and PCOS, they declared variations in the *LEPR* locus has affected the insulin regulation, as a hypothesis (16). Tu et al. (30) suggested that there is no significant association between LEPR R223Q and PCOS in a Chinese population; however, our data demonstrated a protective effect of G allele (R223Q) against PCOS. In addition, a meta-analysis study in Chinese population showed that two variants of *LEPR* (R223Q and P1019P) are related to obesity (31). 

## Conclusion

This is the first report on the relationship of *LEPR* gene variants and PCOS in a population of women from Iran. Here, we showed that R223Q and P1019P of the *LEPR* were risk factors for PCOS susceptibility. This may be useful in biomarker detection and future approaches of PCOS gene therapy. However, it needs to be approved in larger population with different races. 
